# Blocking effect of twin boundaries on partial dislocation emission from void surfaces

**DOI:** 10.1186/1556-276X-7-164

**Published:** 2012-03-02

**Authors:** Lifeng Zhang, Haofei Zhou, Shaoxing Qu

**Affiliations:** 1 Department of Engineering Mechanics, Zhejiang University, Hangzhou, 310027, China; 2 Soft Matter Research Center (SMRC), Zhejiang University, Hangzhou, 310027, China

**Keywords:** twin boundaries, void, dislocations, strength, molecular dynamics simulations

## Abstract

Recent discovery that nanoscale twin boundaries can be introduced in ultrafine-grained metals to improve strength and ductility has renewed interest in the mechanical behavior and deformation mechanisms of these nanostructured materials. By controlling twin boundary spacing, the effect of twin boundaries on void growth is investigated by using atomistic simulation method. The strength is significantly enhanced due to the discontinuous slip system associated with these coherent interfaces. Atomic-scale mechanisms underlying void growth, as well as the interaction between twin boundaries and the void, are revealed in details.

## Introduction

Ultrahigh strength and large elongation to failure can be concurrently achieved in nano-twinned (NT) polycrystals [1-3]. The rate-controlling process governing the plastic deformation in these materials is mediated by coherent twin boundaries (TBs). It is known that TBs not only provide barriers to dislocation motion but also sustain the strain hardening capability of the specimens without early shear localization [4,5].

Voids are crucial point defects that are inevitable during fabrication and deformation of materials. Nanocrystals with voids have many unique properties, including lattice orientation sensitivity [6-8], size-dependent yielding stresses [6,9,10], void volume fraction-dependent elastic modulus [11], and void shape effect on stress concentration [11]. Considerable efforts have been devoted to investigate the nucleation, growth, and coalescence of voids. Specifically, void growth has been proven to be governed by a dislocation-emission-based mechanism [12]. It is found that dislocation loops carrying outward flux of matters are nucleated at void surfaces [9,13]. Simulations performed by Seppala et al. are focused on void coalescence, involving both single and double voids cases [14]. It is revealed that the onset of void coalescence occurs when the intervoid ligament distance reaches approximately one void radius. However, the deformation mechanisms associated with voids in nanostructured crystals with internal boundaries, especially coherent TBs, are yet to be understood.

In the present work, molecular dynamics simulations are performed to investigate the process of void growth in NT crystals. It is shown that the motion of surface dislocations emitted from void defects is strongly confined by surrounding TBs, resulting in higher strengths. Atomistic analysis is performed to illustrate the interaction between TBs and voids.

## Methods

**Figure 1:**
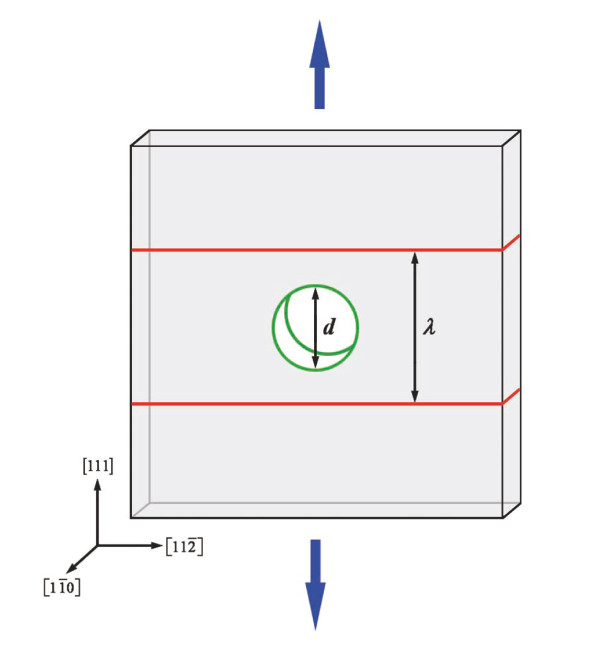
**Schematic of a simulated sample**. The red interfaces represent the TBs, while the green cylinder stands for the central void. *d *and *λ *indicate the diameter of the central void and the TB spacing, respectively. The blue arrows indicate uniaxial tension along the Y [111] direction.

Quasi-three-dimensional crystalline metals, each embedded with a nanometer-sized void in the center, were built for this study. Coherent TBs were inserted in the crystals to investigate their blocking effect on the motion of void surface dislocations. Figure 1 shows the typical geometry of our simulation samples which possesses a dimension of 50 × 50 × 4 nm^3^. Two controllable parameters, i.e., the void size and the TB spacing, are noted as *d *and *λ*, respectively.

The embedded atom method potential [15] for Cu was applied. Periodic boundary conditions were imposed on all three dimensions. The time step was chosen as 1 fs. For the precise control of temperature and pressure, Nose-Hoover thermostat and barostat were adopted [16,17]. The crystal was first annealed at 300 K and 0 pressure for 100 ps to reach equilibrium. After relaxation, uniaxial tension along the Y [111] direction (indicated by the blue arrows in Figure 1) with a constant strain rate of 1 × 10^8 ^s^-1 ^is performed on the crystal during which the stress components, *σ*_xx _and *σ*_zz_, are kept as zero.

Common neighbor analysis [18,19] was used to clarify defects. The coloring scheme for various local structures is as follows: gray for face centered cubic (fcc) atoms, red for hexagonal close packed atoms, and green for atoms with other local crystal structures.

## Results and discussion

### Dependence of yielding stress on void size

**Figure 2:**
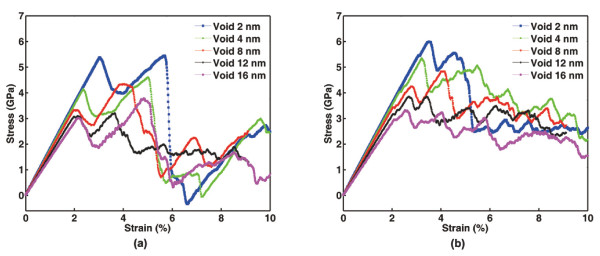
**Typical stress-strain curves of the simulated samples**. (**a**) Twin-free Cu samples and (**b**) NT Cu samples. The diameter *d *of the central void varies from 2 to 16 nm, while the TB spacing is kept at 25 nm.

By controlling the diameter of embedded voids ranging from *d *= 2 to *d *= 16 nm, uniaxial tensile loading up to a maximum strain of 10% was performed on a series of Cu samples, and the corresponding stress-strain curves were obtained. As shown in Figure 2a, after an initial elastic range, the first peak occurs representing yielding through dislocation emission from the void surfaces. It is shown that larger voids lead to earlier yielding of the specimens, consistent with the previous results [9]. It is known that surface stress plays an important role in the plastic deformation of materials with nanoscale defects. The void size dependence of yielding stress in our simulated samples is attributed to the effect of surface stress. Besides, it should also be noted that an increasing void size results in a decreased effective cross-sectional area along the tensile direction, which leads to a higher stress and thus promotes early yielding. The stress drop after yielding on each curve is a result of free dislocation propagation.

### TB strengthening behavior

To demonstrate the blocking effect of twin planes, another set of tension simulations were performed on Cu samples with various void sizes but this time, two parallel twin planes with a spacing of *λ *= 25 nm were added into each sample. As shown in Figure 2b, the samples deform elastically at small strains. Sudden strain excursions associated with surface dislocation nucleation are observed, indicating the onset of yielding, at stresses about the same level as those corresponding to the yielding peaks in Figure 2a. Upon further loading, the stresses continue to rise with the increasing strain, in contrast to the stress drops observed in Figure 2a after the emergence of the first stress peaks, implying that the dislocation propagation may be hindered by the twin planes nearby.

**Figure 3:**
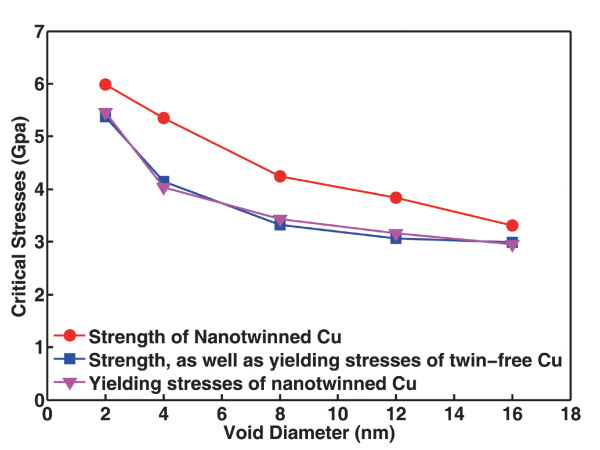
**The relation between critical stresses and void size**. Data points are extracted from the stress-strain curves in Figure 2.

The above observations are summarized in Figure 3. Here, strength is defined as the stress at which the first stress peak occurs. For twin-free Cu samples, strength is equivalent to the yielding stress of the material. For the case of NT Cu samples, however, yielding stress (when strain excursions begin to occur) is lower than the strength due to the presence of twin planes. As shown in Figure 3, the close agreement between the blue (yielding stresses of twin-free samples) and pink (yielding stresses of NT samples) data implies that twin planes do not influence the nucleation of dislocations from void surfaces. However, the deviation in the strength of the two materials due to the introduction of twin planes is clearly discernable (see the separation between blue and red data), indicating that twin planes exhibit strengthening behavior by blocking dislocation motion. Detailed analysis about TB strengthening will be performed in the following sections.

### Mechanisms of yielding and TB strengthening

**Figure 4:**
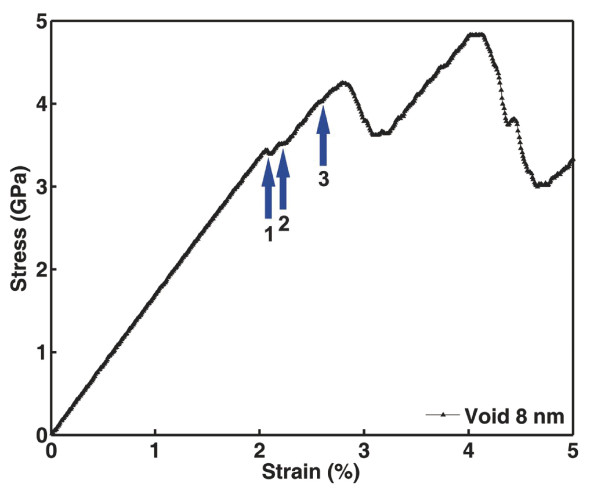
**The stress-strain curve of an NT Cu sample**. Three individual strain excursions are labeled as 1, 2, and 3.

**Figure 5:**
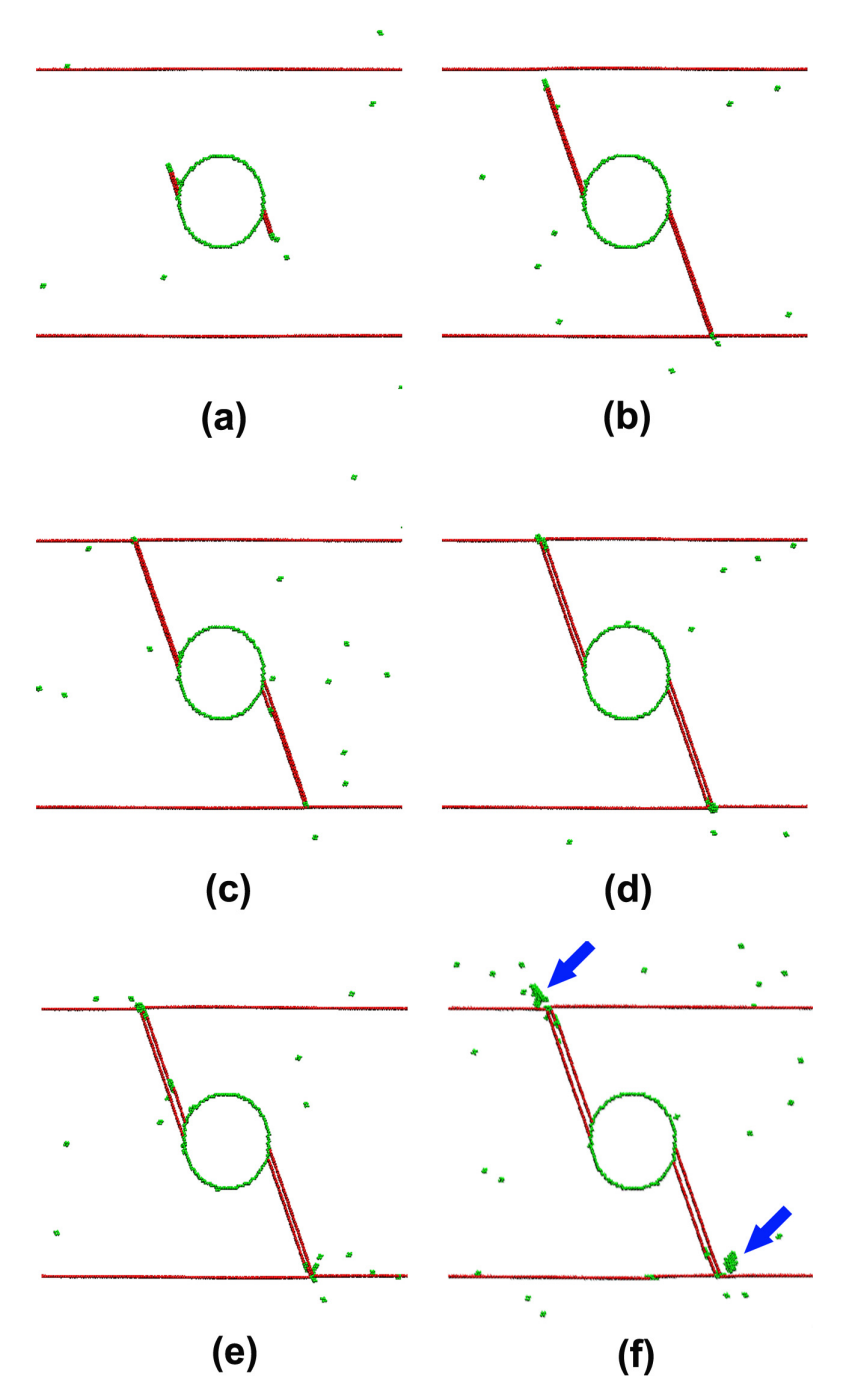
**Atomic-scale snapshots showing deformation details relating to the three strain excursions shown in Figure 4**. Here, fcc atoms are not shown for clarity. (**a**) Partial dislocations are nucleated at the void surface. (**b**) Dislocation motion is hindered by the TBs, leaving TB steps on the twin planes and intrinsic stacking fault ribbons. **(c) **Subsequent partial dislocations are nucleated from the same location at the void surface but on adjacent atomic planes. (**d**) TB blocking dislocation motion, leaving TB steps and extrinsic stacking faults, (**e**) Subsequent dislocation emission on adjacent atomic planes. (**f**) The formation of twin nuclei due to TB blocking. The blue arrows indicate the nucleation of dislocations from TB steps.

In this section, we investigate the atomistic mechanisms of the microstructural evolution during the deformation of the Cu samples. For brevity, the NT Cu sample containing a void 8 nm in diameter was selected for detailed atomic-scale analysis. Figure 4 shows the stress-strain curve of this Cu sample. Three individual strain excursions, marked with Arabic numerals 1, 2, and 3, are visible on the curve before the stress approaches the first peak. Each strain excursion occurs by the nucleation of surface dislocations from the void and ceases by the interactions between the dislocations and the twin planes. As shown in Figure 5a, Shockley partial dislocations of type $\frac{1}{6}\left\langle 112\right\rangle$ are nucleated on the opposite sides of the void due to geometrical symmetry, causing a sudden increase in the macroscopic strain. The motion of these lattice defects into the crystal leaves ribbons of stacking faults and finally meets the preexisting twin planes, as shown in Figure 5b. Twin planes are effective barriers to dislocation motion, which is reflected by the elimination of the first strain excursion on the stress-strain curve. Upon further deformation, emission of partial dislocations from the same location of the void surface but on adjacent {111} planes is observed, as shown in Figure 5c. This leads to the second strain excursion on the stress-strain curve, as well as the formation of extrinsic stacking faults, as shown in Figure 5d. The motion of these dislocations, again, is blocked by the twin planes leading to a regain in material strength. As shown in Figure 5e and 5f, the third strain excursion occurs along with a deformation process similar to the previous ones, but it has led to the formation of a two-layer twin nucleus.

A lot of efforts have been devoted to understand the mechanisms of TB interactions with different types of dislocations [2,5,20,21]. A detailed discussion on this topic is not within the scope of this paper. We emphasize here that the strength of the NT material, which is reflected by the first peaks on the stress-strain curves in Figure 2b, is actually determined by the strength of the twin planes against dislocation penetration. This is demonstrated by Figure 5f, which is captured on the occasion of the first peak of the stress-strain curve. At that time, there is an obvious sign of dislocation nucleation around the TB segments intersected by the deformed twin nucleus, leading to slipping either in the crystal or along the twin planes (indicated by arrows in Figure 5f). These dislocation activities clearly indicate that the twin planes have reached their limitation of strengthening. In other words, dislocation barriers have become dislocation sources since then. The sudden stress drops in Figure 2b is another evidence of this transition.

### Dependence of void yielding and sample strength on TB spacing

**Figure 6:**
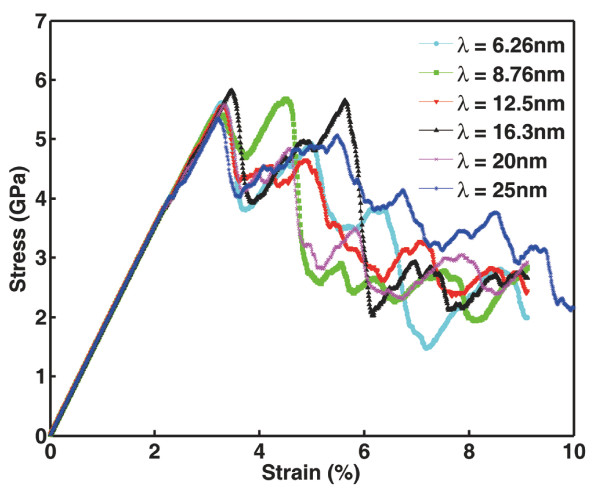
**Stress-strain curves of NT Cu samples with various TB spacing**. A void 4 nm in diameter is embedded in all NT Cu samples with TB spacing *λ *varying from 6.26 to 25 nm.

**Figure 7:**
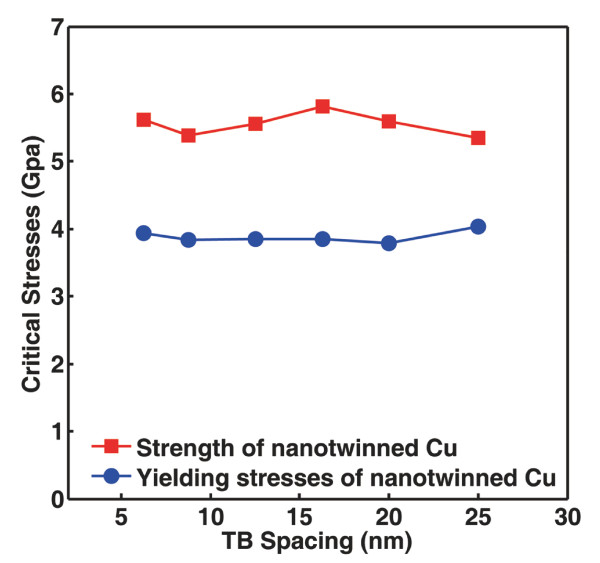
**The effect of TB spacing on critical stresses**. Data points are collected from the stress-strain curves in Figure 6.

Previous reports [2,5,20] have shown that mechanical properties of NT materials are closely related to the width of twin lamellae also termed as TB spacing. Reducing TB spacing into the nanometer scale is capable of altering mechanisms of plastic deformation causing either strengthening or softening of NT materials [2,20]. It is thus interesting to see how the strength of the NT sample in the present work, particularly embedded with a central void, changes with TB spacing. Uniaxial tensile loading procedure is the same. The central void is fixed to be 4 nm in diameter, while TB spacing is controlled to alter from 6.26 to 25 nm. Figure 6 shows the stress-strain curves of the NT samples with various TB spacing. For the sake of clarity, we compare the critical stresses of the five simulated samples in Figure 7. As a highly coherent interface, TB produces neither modulus mismatch nor coherency stress in the neighboring lattice. Changing TB spacing does not affect the local stress field nearby the void. Thus, the yielding stress required for dislocation nucleation from the void is not sensitive to TB spacing. Moreover, for NT metals with a specified volume, reducing TB spacing is equivalent to increasing the density of TBs, which imposes a stronger resistance against lattice slipping. In other words, TB strengthening is a collective behavior of a large number of TBs suppressing dislocation activities. In our simulations, however, TB density is fixed (two TBs in each sample) as the TB spacing decreases. Sample strength is actually determined by the strength of the two TBs surrounding the void. Reducing TB spacing only affects the slip distance of the dislocations before they interact with the TBs, not the inherent strength of a TB. Therefore, the sample strength is not sensitive to TB spacing.

## Conclusions

The above results make it clear that the yielding stress of a crystalline metal embedded with nanoscale voids depends mainly on the size of these voids but much less on the surrounding coherent TBs. The strength of a Cu sample, on the other hand, can be significantly enhanced by introducing coherent TBs into the crystal. It is also shown that the intrinsic strength of an individual twin plane imposes limitation on the blocking effects of the embedded TBs. The influence of the spacing between two twin planes, however, seems to be less significant, as demonstrated in Figure 7. It is worth noting that although the change in TB spacing leads to unnoticeable fluctuations in materials strength, further investigation is still required to understand the underlying atomistic mechanisms especially in samples with larger dimensions and higher densities of nanoscale voids.

## Abbreviations

NT: nano-twinned; TBs: twin boundaries.

## Competing interests

The authors declare that they have no competing interests.

## Authors' contributions

All authors contributed equally to this work. SQ and HZ conceived the project. LZ performed the simulations. All authors analyzed data, discussed the results, and wrote the paper. All authors read and approved the final manuscript.
